# Religion and Spirituality as Social Determinants of Sleep Health Across the Globe: A Narrative Review

**DOI:** 10.1007/s40675-026-00374-y

**Published:** 2026-05-19

**Authors:** Rupsha Singh, Symielle A. Gaston, Harold G. Koenig, Chandra L. Jackson

**Affiliations:** 1 Epidemiology Branch, National Institute of Environmental Health Sciences, National Institutes of Health, Department of Health and Human Services, Research Triangle Park, NC USA; 2 Department of Psychiatry and Behavioral Sciences and Department of Medicine, Duke University Medical Center, Durham, NC USA; 3 Intramural Program, National Institute on Minority Health and Health Disparities, National Institutes of Health, Department of Health and Human Services, Bethesda, MD USA; 4 Present Address: 111 TW Alexander Drive, MD A3-05, Research Triangle Park, NC 27709 USA

**Keywords:** Religion, Spirituality, Sleep, Sleep duration, Sleep quality, Insomnia

## Abstract

**Purpose of review:**

To summarize existing evidence of associations between religion and spirituality in relation to multiple dimensions of sleep health, illuminate strengths and limitations of the existing literature, and outline gaps that inform opportunities for future research.

**Recent findings:**

Positive dimensions of religion and spirituality, such as spiritual well-being, supportive coping, and community integration, were generally associated with better sleep outcomes across global populations. Adverse experiences, including doubt, conflict, and spiritual struggle, were often associated with poorer sleep. Among the 59 included studies, most were cross-sectional and relied on self-reported measures, used inconsistent conceptualizations of religion and spirituality, did not sufficiently assess potentially adverse religious or spiritual experiences, and were limited to certain geographic regions of the world (e.g., North America and Southwest Asia).

**Summary:**

Positive dimensions of religion and spirituality (e.g., meaning, coping) were generally associated with better sleep, while struggles and doubt were associated with poorer sleep. Future research that employs longitudinal and experimental designs, validated multidimensional measures, objective sleep assessments, and diverse populations is needed to clarify mechanisms and inform culturally responsive approaches to promoting sleep health.

**Supplementary Information:**

The online version contains supplementary material available at 10.1007/s40675-026-00374-y.

## Introduction

Sleep is increasingly recognized as a multidimensional determinant of health, encompassing duration, efficiency, timing, regularity, and satisfaction/quality [1] yet, healthy sleep is not experienced uniformly across populations [2]. Sleep disturbances are associated with myriad health outcomes, including associated with myriad health outcomes, including but not limited to cardiometabolic disease, mental health problems, impaired cognition, reduced quality of life, and premature mortality [3–5]. Although sleep is foundational to health across the entire life course, from early development through older adulthood, these consequences are particularly salient in midlife and older adulthood, when age-related changes in sleep architecture and circadian regulation increase vulnerability to sleep disturbances. With advancing age, individuals experience reductions in slow-wave sleep, increased nocturnal awakenings, greater sleep fragmentation, and a tendency toward earlier chronotype [6]. The prevalence of insomnia symptoms and sleep-disordered breathing also rises in older adulthood [6]. These normative and pathological changes underscore the importance of identifying psychosocial, behavioral, and cultural factors that may either buffer or exacerbate sleep disruption across the lifespan.

Although understudied, religion and spirituality are central constructs in understanding these influences. Religion has been defined as an organized system of beliefs, practices, rituals, and symbols designed to bring individuals closer to the sacred or transcendent, and to foster an understanding of individuals’ relationship and responsibility to others in a community [7, 8]. Spirituality, in contrast, is broader and refers to the aspect of humanity that involves seeking and expressing meaning and purpose, and experiencing connectedness to self, others, nature, or the sacred [9]. These constructs overlap yet remain distinct: religiosity typically reflects external practices such as service attendance or prayer, while spirituality captures inward experiences of transcendence and meaning. Both positive and negative dimensions of religion and spirituality (R/S) may influence health. For instance, positive engagement, such as prayer, meditation, or participation in regular religious community activities, can provide coping resources, foster social support, and encourage healthy routines, whereas doubt, conflict, or spiritual struggle may exacerbate distress and disrupt well-being [7, 8].

Religion and spirituality are not static across the life course but instead demonstrate meaningful developmental patterns beginning in early childhood and continuing through older adulthood [10]. Religious and spiritual socialization often begins in early life through family practices, communal worship, and ritual participation, with children and adolescents actively engaging in prayer, religious language (e.g., Arabic; Hebrew; Sanskrit), and faith-based routines [11]. Across many cultural contexts, levels of religious service attendance, private prayer, and the perceived importance of faith often shift across adulthood, with some studies showing increases in religious involvement and reliance on religious coping in midlife and older adulthood, while religious doubt and questioning may decline [12, 13]. Older adults are also more likely than younger adults to draw upon religious coping in response to health challenges, bereavement, and role transitions, suggesting that R/S may become increasingly central as individuals confront age-related stressors and existential concerns [12, 14, 15]. At the same time, cohort effects, secularization trends, and differences in religious socialization mean that patterns of engagement vary across generations [12]. Importantly, these developmental shifts in R/S may intersect with normative changes in sleep architecture and circadian rhythms across the lifespan. For example, increased reliance on spiritual coping in later life may buffer stress-related hyperarousal that contributes to insomnia, whereas spiritual struggle or rigid ritual demands could exacerbate nighttime distress in the context of already fragmented sleep. Despite these theoretical intersections, relatively few studies explicitly examine age as a moderator of R/S–sleep associations, representing a notable gap in the literature.

Investigating R/S in relation to sleep is important for several reasons. First, R/S are widely observed worldwide, with 75.8% of the global population identifying with a religion [16] and 72% believing in God [17], underscoring the extent to which religious practices function as culturally embedded resources that may shape sleep-related behaviors and experiences. Second, R/S can influence psychological, behavioral, and biological pathways directly tied to sleep, including stress regulation, bedtime routines, substance use, circadian alignment (e.g., through structured religious practices, such as morning prayer that advances wake timing and light exposure, or nighttime rituals and observances that may delay bedtime or reduce sleep opportunity), and inflammatory processes [18]. For instance, Do and colleagues (2025) demonstrated that even subtle changes in sleep duration causally influence gratitude, flourishing, and prosocial behaviors, outcomes that closely parallel the positive psychological resources often fostered through religious and spiritual engagement [19]. Third, R/S involvement is often greater among women and many racially and ethnically diverse populations, groups that also report more sleep problems, raising the possibility that R/S may play a unique role in sleep outcomes in these populations [20–22]. Finally, integrating R/S into sleep research offers opportunities to expand prevention and intervention strategies beyond traditional biomedical and behavioral approaches.

The objective of this review is to summarize the available literature on R/S and sleep health, extending prior research. For instance, the only previous review by Hill et al. (2018) summarized seven U.S. population-based studies and proposed an initial conceptual model suggesting that religious involvement may support healthier sleep through reduced psychological distress, substance use, and physiological arousal [18]. However, the review focused only on religious involvement, did not examine spirituality or negative religious experiences, and did not include assessment of associations with multiple dimensions of sleep health or examine global populations. To address these gaps, we provide an updated and expanded summary, highlight key conceptual and methodological considerations, and propose directions for future research.

## Methods

### Literature Search

This narrative review aimed to summarize evidence on the associations between R/S and sleep health. A narrative approach was warranted given the heterogeneity of R/S constructs and sleep measures and the limited number of directly comparable studies that preclude a systematic review or meta-analysis. Although narrative in scope, articles were identified using a structured search strategy. Searches were conducted in PubMed, Web of Science, and Embase between January 2025 and December 2025. Search terms were developed based on the exposure and outcome of interest. A comprehensive set of librarian-generated search terms is provided in Supplemental Table 1. Reference lists of eligible papers and relevant reviews were also manually screened to identify additional studies.

### Eligibility Criteria

Publications eligible for inclusion were peer-reviewed, empirical studies of original research, reviews, and meta-analyses that examined religion, spirituality, or specific religious practices in relation to sleep health or sleep disorders. Only articles published in English between January 2000 and December 2025 were considered. Studies were excluded if religion or spirituality was treated solely as a background covariate without reporting associations with sleep, if sleep was not a primary or secondary outcome, or if the work was limited to abstracts, case reports or non–peer-reviewed sources.

### Data Extraction and Synthesis

Titles and abstracts of all records were screened for relevance. Full texts were reviewed to determine final eligibility. For each study, data were extracted on study design, population characteristics (including age, sex/gender, race and ethnicity, geographic region, and religious affiliation when reported), sample size, measures of R/S, sleep outcomes assessed, and principal findings. To contextualize the study, race and ethnicity data were extracted and reported whenever available, reflecting prior evidence of racial and ethnic differences in R/S engagement and in sleep health [22, 23]. Evidence was synthesized narratively, grouped by dimensions of sleep health (duration, quality, insomnia symptoms, disturbances, and objective measures).

## Results

### Study Characteristics

A total of 59 studies published between 2004 and 2025 met inclusion criteria (Table 1). Sample sizes ranged from 13 to 26,742 participants. All studies were conducted among adults, spanning young adulthood through older adulthood (approximately ages 18 to late 80 s). Most studies included participants of both sexes (*n* = 42), while a small proportion focused exclusively on women (*n* = 7) or men (*n* = 1); the remaining studies did not report sex-specific composition or were review-based. Furthermore, most investigations employed cross-sectional designs (*n* = 34) while a smaller proportion used interventional approaches, including randomized controlled trials and quasi-experimental studies (*n* = 13). The remaining studies included qualitative designs (*n* = 3), scoping or narrative reviews (*n* = 4), systematic reviews and meta-analyses (*n* = 3), and a limited number of longitudinal (*n* = 1) or mixed-methods studies (*n* = 1). Geographically, research was heavily concentrated in the United States (U.S.), which accounted for nearly half of all included studies (*n* = 28, 47%). Additional studies were conducted in Brazil, Canada, Iran, the United Arab Emirates, Germany, Israel, Turkey, Indonesia, Taiwan, China (including Hong Kong), South Korea, and India. No studies were identified from the African region, and relatively few were conducted in Latin America or South Asia, reflecting a notable geographic imbalance in the literature.

**Table 1:** Summary of study characteristics and findings on religiosity/spirituality and various dimensions of sleep health

Author & Year	Study location	Study design	Sample	Religion	Religiosity/Spirituality	Sleep outcome measurement	Positive findings	Negative findings
Sleep duration								
Margolis & Reed, 2004 [24]	United Arab Emirates	Cross-sectional	*N* = 137 (Men = 28%; Women = 72%) pre-Ramadan, *N* = 126 (Men = 27%, Women = 73%) during Ramadan, *N* = 109 (Men = 28%, Women = 72%) post-Ramadan; Muslim medical students; Age range = 19–23 years	Muslim	Religious practices during Ramadan, including fasting, altered sleep-wake schedules, and pre-dawn prayers	Self-reported sleep duration	None reported	Night sleep duration significantly decreased during Ramadan (*p* < 0.001), and total sleep duration declined compared to pre-Ramadan levels (*p* = 0.04).
Zarhin, 2023 [25]	Israel	Qualitative	*N* = 31; Midlife Israeli Muslim adults (*n* = 18) and Jewish adults (*n* = 13) who identified as religious or very religious; Men (*n* = 14), Women (*n* = 17); Age: M = 49.41 years	Muslim and Jewish	Religious observance, including prayer times, beliefs, and prescribed sleep positions (qualitative study; no specific instrument used)	Self-reported sleep duration (qualitative interviews)	None reported	Adherence to prescribed prayer times disrupted sleep duration, particularly for Muslims and Jewish men, who had to wake up early but could not always go to sleep early enough.
Johnson et al., 2023 [26]	United States	Cross-sectional	*N* = 3,711; 53% women; Within racial and ethnic groups, 53% White women, 52% Hispanic women, 56% Black women; Mean age ± SE for total sample was 57 ± 0.3 years; Within racial and ethnic groups, 55 ± 0.6 years among Black adults, 57.9 ± 0.4 years among Hispanic adults, 51.9 ± 1.1 years among White adults	Christian	Church or religious attendance (measured as weekly attendance vs. less than weekly)	Sleep duration (self-reported sleep hours per weekday)	Church attendance was associated with lower prevalence of short sleep duration in unadjusted models.	In fully adjusted models, the association between church attendance and sleep duration was attenuated and no longer significant.
Goerge et al., 2024 [27]	United States	Cross-sectional	*N* = 323; African American long-term breast cancer survivors; Age: M = 54.8 years (SD = 9.89 years)	Not reported	Spirituality, measured using the Functional Assessment of Chronic Illness -Therapy Spiritual Well-Being 12 Item Scale (FACIT-Sp-12)	Pittsburgh Sleep Quality Index (PSQI) and self-reported questionnaire data	Higher spirituality scores were associated with longer sleep duration (> 7 h per night) (aOR: 1.72, 95% CI: 1.05–2.83). The meaning, peace, and faith subscales were all positively associated with sleep duration.	None reported
Sleep quality								
Yang et al., 2008 [28]	Taiwan	Cross-sectional	*N* = 861; Hemodialysis patients from 14 urban dialysis clinics; Good sleepers: Male (48.4%), Women (51.6%); Poor sleepers: Male (42.3%), Women (57.7%); Age : Good sleepers M = 55.8 years (SD = 13.7) years; Poor sleepers M = 58.1 years (SD = 13.1 years)	Not reported	Spiritual and religious activity, measured using the Royal Free Questionnaire (RFQ)	Sleep quality, measured using the Pittsburgh Sleep Quality Index (PSQI)	Patients who exercised religious beliefs more strongly reported less trouble with daytime dysfunction.	Patients with stronger spiritual beliefs reported more sleep disturbances. There was no significant correlation between global PSQI scores and total religious/spiritual activity scores.
Ellison et al., 2011 [29]	United States	Cross-sectional	*N* = 1,250; Nationwide sample of Presbyterian Church (USA) members (race and ethnicity not provided); Age: M = 59.47 years (SD = 13.48 years)	Not reported	Religious doubts, measured using a three-item index assessing doubts about faith due to evil in the world, faith-science conflicts, and meaning in life	Sleep quality, measured using three items from the Pittsburgh Sleep Quality Index (PSQI) assessing overall sleep quality	None reported	Religious doubts were significantly associated with poorer self-rated sleep quality. These associations were partially mediated by psychological distress.
Eslami et al., 2014 [30]	Iran	Cross-sectional	*N* = 190; Hemodialysis patients in Isfahan, Iran; Male (60.4%), Female (39.6%)	Not reported	Spiritual well-being, measured using the Ellison and Paloutzian Spiritual Well-Being Scale (SWBS)	Sleep quality, measured using the Pittsburgh Sleep Quality Index (PSQI)	A significant relationship was found between spiritual well-being and sleep quality (*P* < 0.04, *r* = 0.149), suggesting that higher spiritual well-being was associated with better sleep quality.	None reported
Khoramirad et al., 2015 [31]	Iran	Cross-sectional	*N* = 80; Shite Muslim women with breast cancer; Female (100%); Age: M = 56.14 years (SD = 9.28 years)	Not reported	Spiritual well-being, measured using the Spiritual Well-Being Scale (SWBS), and religious activities, measured using the Religious Activities (RA) questionnaire	Sleep quality, measured using the Pittsburgh Sleep Quality Index (PSQI)	Stronger beliefs in God’s support and a sense of purpose in life were associated with better sleep quality. Attendance in mosque or religious places was negatively correlated with PSQI scores, indicating better sleep quality.	Overall spiritual well-being and religious activities were not significantly correlated with global PSQI scores. However, religious activity was positively correlated with sleep latency and the use of sleep medications, suggesting that individuals with poor sleep may engage more in religious activities.
Krause & Ironson, 2017 [32]	United States	Cross-sectional	*N* = 1,774; Nationally representative adult sample; Male (38.1%), Female (62%); Age: M = 52.8 years (SD = 18.9 years)	Not reported	Church attendance, spiritual support, God-mediated control, hope (measured using self-reported survey items)	Sleep quality, measured using two items from the Pittsburgh Sleep Quality Index (PSQI)	Greater spiritual support was associated with stronger God-mediated control beliefs, which in turn were linked to greater hope. Hope was directly associated with better sleep quality.	Church attendance, spiritual support, and God-mediated control did not have direct effects on sleep quality but were indirectly linked through hope.
Yun et al., 2017 [33]	South Korea	Randomized Controlled Trial	*N* = 46; Breast cancer survivors; Female (100%); Age: M = 48.44 years (SD = 8.16 years)	Not reported	Mind Subtraction Meditation (MSM) – a spiritual meditation intervention	Sleep quality measured using the Korean Pittsburgh Sleep Quality Index (PSQI-K)	Participants in the MSM group showed significant improvements in sleep quality compared to the self-management education (SME) group (*p* = 0.010).	None reported
Hill et al., 2018 [18]	United States	Scoping Review	Review article (7 population-based studies reviewed)	Not reported	Religious involvement (measured by religious attendance, private religious practices, God-mediated control, sacred body views, and religious struggles)	Multiple sleep dimensions reviewed (sleep duration, overall sleep quality, sleep initiation, and use of sleep medications)	Religious involvement was generally associated with better sleep outcomes across multiple studies, with secure attachment to God, assurance of salvation, and a sacred body view being protective for sleep quality.	Religious doubts were negatively associated with sleep quality, leading to more sleep disturbances and increased use of sleep medications.
Knowlden et al., 2018 [34]	United States	Cross-sectional	*N* = 200; University students in entry-level development course (82.5% White, 12.5% African American, other not specified); Female (56%), Male (44%); Age: M = 19.98 years (SD = 1.54 years)	Not reported	Spiritual beliefs, measured using the Beliefs and Values Scale (BVS)	Sleep quality, measured using the Pittsburgh Sleep Quality Index (PSQI)	Higher spiritual beliefs were associated with lower psychological distress and better sleep quality. Individuals with low distress had significantly stronger spiritual beliefs and better sleep quality.	Women reported lower sleep quality than men, but there was no significant difference in spiritual beliefs between sexes.
Ellison et al., 2019 [35]	United States	Cross-sectional	*N* = 1,410; Nationally representative sample from the 2017 Baylor Religion Survey (race and ethnicity not provided); Male (47%), Female (53%); Age: M = 49.16 years (SD = 17.65 years)	Not reported	Religious attendance, private religiousness (prayer, scripture reading), secure attachment to God, assurance of salvation (measured using self-reported survey items)	Self-reported past-month sleep quality (composite index based on sleep duration, trouble falling asleep, and feeling rested in the morning)	Secure attachment to God and assurance of salvation attenuated the negative association between stressful life events and sleep quality. Depressive symptoms mediated these effects.	Religious attendance and private religiousness (prayer and scripture reading) did not significantly buffer the effects of stressful life events on sleep quality.
Purwanti et al., 2019 [36]	Indonesia	Quasi Experimental Pilot Study	*N* = 60; Muslim patients with chronic kidney failure undergoing hemodialysis; Control group: Male (60%), Female (40%), Age: 17–25 years (3.3%), 26–45 years (33.3%), 46–65 years (63.3%); Intervention group: Male (43.3%), Female (56.7%), Age: 17–25 years (0%), 26–45 years (30%), 46–65 years (70%)	Not reported	Religious relaxation therapy (spiritual-based relaxation based on Islamic teachings)	Sleep quality measured using the Pittsburgh Sleep Quality Index (PSQI)	Participants in the intervention group showed significant improvements in sleep quality after religious relaxation therapy compared to the control group (*p* = 0.000).	None reported
Moradi et al., 2022 [37]	Iran	Randomized Controlled Trial	*N* = 40; Pregnant women in Abhar, Iran, Women (100%); Mean age intervention group = 26.65 years, Mean age control group = 28.55 years	Muslim	Spiritual content counseling based on Islamic teachings (8-session intervention twice a week for 4 weeks)	Sleep quality measured using the Pittsburgh Sleep Quality Index (PSQI)	Participants in the intervention group showed significant improvements in sleep quality compared to the control group. The effects persisted through the second and third trimesters (*p* < 0.001).	None reported
Nguyen et al., 2022 [38]	United States	Cross-sectional	*N* = 459 older African American adults (55 + years) from the National Survey of American Life-Reinterview; Men (40.56%), Women (59.44%); Age: M = 65.97 years (SD = 8.19 years)	Not reported	Religious service attendance, reading religious texts, watching religious television programs, listening to religious radio programs, prayer, and subjective religiosity (self-reported survey measures)	Restless sleep (using an item from the Center for Epidemiologic Studies Depression Scale (CES-D) and sleep satisfaction (self-reported survey response)	Religious service attendance (less than once a year, at least once a week, or nearly every day) was associated with greater sleep satisfaction.	Watching religious television programs was associated with more restless sleep. Subjective religiosity was negatively associated with sleep satisfaction.
Yousofvand et al., 2023 [39]	Iran	Randomized Controlled Trial	*N* = 117; Muslim stroke patients with no sleep disorder before stroke; Intervention group: Female (45%), Male (55%), Age – M = 56.58 years, SD = 12.89 years; Control group: Female (53.3%),Male (46.7%), Age – M = 56.15 years (SD = 12.86 years)	Muslim	Spiritual care program, measured using the Spiritual Well-Being Scale (SWBS)	Sleep quality, measured using the Pittsburgh Sleep Quality Index (PSQI)	Patients in the intervention group showed significant improvements in sleep quality compared to the control group (*p* < 0.001).	None reported
Zarhin, 2023 [25]	Israel	Qualitative	*N* = 31; Midlife Israeli Muslim adults (*n* = 18) and Jewish adults (*n* = 13) who identified as religious or very religious; Men (*n* = 14), Women (*n* = 17); Age: M = 49.41 years	Muslim and Jewish	Religious observance, including prayer times, beliefs, and prescribed sleep positions (qualitative study; no specific instrument used)	Self-reported sleep quality (qualitative interviews)	Participants believed that deep faith in God and bedtime prayers helped reduce stress, promoting better sleep quality.	Some Muslim adults reported that missing prayers led to guilt, which in turn negatively affected sleep quality.
Alaei et al., 2024 [40]	Iran	Randomized Clinical Trial	*N* = 90; Male prisoners admitted to the emergency department of the central prison clinic; Male (100%); Age: M = 35 years (SD = 7.01 years)	Muslim	Spiritual care intervention and aromatherapy with a focus on Islamic teachings	Sleep quality, measured using the Pittsburgh Sleep Quality Index (PSQI)	Patients receiving spiritual care and aromatherapy showed significant improvements in sleep quality compared to the control group (*p* < 0.05), with reductions in sleep latency and overall better sleep efficiency.	None reported
Khalili et al., 2024 [41]	Iran	Cross-sectional	*N* = 222; Cancer patients in Hamadan, Iran; Female (55.4%), Male (44.6%); Age < 40 years (27.5%), Age > 40 years (72.5%)	Muslim	Spiritual health, measured using the Islamic Spiritual Health Questionnaire (ISHQ)	Sleep quality, measured using the Pittsburgh Sleep Quality Index (PSQI)	None reported	No significant correlation was found between spiritual health and sleep quality (*r* = 0.032, *p* > 0.05). However, anxiety was negatively correlated with sleep quality (*r* = −0.146, *p* < 0.05), suggesting that higher anxiety levels were associated with poorer sleep quality.
Gautama et al., 2025 [42]	Meta-analysis of studies across Asia and North America	Meta-analysis	Meta-analysis of 15 RCTs, *N* = 1,635 cancer patients	Not reported	Managing Cancer and Living Meaningfully (CALM) Therapy, addressing spiritual well-being	Sleep quality (standardized mean difference across studies)	CALM therapy significantly improved sleep quality (SMD = − 1.56) and spiritual well-being.	None reported
Lee et al., 2025 [43]	United States	Scoping Review	Scoping review of 4 studies; midlife African American women (aged 40–65 years)	Not reported	Spirituality and religious beliefs considered in intervention design	Sleep quantity, quality, and insomnia symptoms (literature review)	Faith and spirituality identified as culturally relevant facilitators in tailored sleep interventions.	None reported
Lin et al., 2025 [44]	Taiwan	Cross-sectional	*N* = 376 nurses at a medical center; Male (4%), Female (96%); Age: M = 31.52 years (SD = 7.97 years)	Not reported	Spiritual health measured using the Spiritual Health Scale - Short Form (SHS-SF)	Sleep quality using the Pittsburgh Sleep Quality Index (PSQI)	None reported	The poorer the sleep quality, the lower the spiritual health (*r* = − 0.217, *p* < 0.01).
Longo-Silva et al., 2025 [45]	Brazil	Cross-sectional	*N* = 5,520 adults aged 18–65 from the Sonar-Brazil Survey; Female (69.86%), Male (30.14%); Age: M = 36.43 years (SD = 12.37 years)	Not reported	Religious service attendance (self-reported frequency)	Sleep quality and sleep disorders using the Pittsburgh Sleep Quality Index (PSQI)	Higher religious attendance was associated with better sleep quality and fewer sleep disorders. Effects were mediated by lower depression, smoking, and screen time.	None reported
Wheeler et al., 2025 [46]	United States	Cross-sectional; secondary data analysis from participants of three different studies (genetic trial; adults with co-occurring PTSD; medication study among individuals with AUD and healthy controls; genetics)	*N* = 158; From first study (*n* = 13); Second study (*n* = 130); Third study (*n* = 15); 32.3% Women, 67.7% Men; Age = 43.8 years (SD = 12.0 years); Non-Hispanic African American/Black (46.2%), Non-Hispanic White (39.9%)	Not reported	God consciousness and formal practices (meditation, attendance, etc.), measured using the Religious Behavior and Background (RBB) Questionnaire	Sleep quality (duration, latency, disturbance, etc.), measured using the Pittsburg Sleep Quality Index (PSQI)	None reported	Formal religious practices moderated the association between sexual trauma and alcohol use, which was significantly associated with sleep quality, meaning those who consumed more alcohol reported poorer sleep quality. Experiencing sexual trauma contributed to greater alcohol use, which was linked to worse sleep quality only among those reporting high levels of formal religious practices and was not significant at average or low levels of religious practices.
Azami-Aghdash et al., 2025 [47]	Iran	Systematic review & meta-analysis	53 Iranian studies (clinical trials + quasi-experimental) of spiritual health-based interventions in patients	Islam	Spiritual health-based interventions/spiritual care (varied programs, sessions, duration)	Sleep quality measured using various scales, including the Pittsburg Sleep Quality Index (PSQI).	Spiritual health–based interventions were associated with improved sleep quality.	None reported
Setiyowati et al., 2025 [48]	Indonesia	Quasi-experimental (pretest–posttest control group)	*N* = 44 hypertensive patients; Indonesian Muslim adults; Male 46.1%, Female 53.8%; Mean age 53.1 years	Islam	Benson’s spiritual relaxation and lavender aromatherapy	Sleep quality measured using the Pittsburg Sleep Quality Index (PSQI)	The intervention significantly improved sleep quality and reduced anxiety and blood pressure.	None reported
Wijayanti et al., 2025 [49]	Indonesia	Randomized controlled trial	*N* = 54 hemodialysis patients; Muslim adults; Male ~ 59%, Female ~ 41%; Age: M = ~46 years	Islam	Dhikr Asmaul Husna + salawat (an Islamic audio spiritual therapy)	Sleep quality measured using the Pittsburg Sleep Quality Index (PSQI)	A significant improvement in sleep quality and anxiety was observed in the intervention vs. control.	None reported
Bazrafshan et al., 2025 [50]	Iran	Randomized clinical trial	*N* = 40 postmenopausal women; Iranian Muslim; Female (100%); Age: M = ~ 59 years	Islam	Group spiritual reminiscence therapy	Sleep quality measured using the Pittsburg Sleep Quality Index (PSQI)	Spiritual reminiscence significantly improved global and domain-specific sleep quality.	None reported
Fan et al., 2025 [51]	Global (multiple countries)	Systematic review and meta-analysis of RCTs	18 RCTs; perimenopausal and postmenopausal women	Not reported	Mind–body therapies (yoga, mindfulness, qigong, tai chi, dance)	Sleep quality measured using the Pittsburg Sleep Quality Index (PSQI), Insomnia Severity Index (ISI) and Women’s Health Initiative Insomnia Rating Scale (WHIIRS)	Mind–body therapies significantly improved sleep quality and reduced depression and anxiety compared with controls.	None reported
Basis et al., 2025 [52]	Israel	Cross-sectional	*N* = 233; Druze adults; religious (*n* = 93), non-religious (*n* = 140); Male 28.5%, Female 71.5%; Age: M = 36.6 years (SD 10.8 years)	Druze Faith	Religiosity and religious orientation (end, means, quest)	Self-reported s sleep duration and quality of sleep.	Higher religiosity associated with better sleep quality; anxiety mediated associations.	A “religion as quest” orientation was associated with poorer sleep.
Yousofvandet al., 2025 [53]	Iran	Randomized controlled trial	*N* = 95 hemodialysis patients; Iranian Muslim; Male ~ 40%, Female ~ 60%; Age: M = ~ 42 years	Islam	Spiritual care program	Sleep quality measured using the Pittsburg Sleep Quality Index (PSQI)	Spiritual care significantly improved sleep quality vs. control.	None reported
Insomnia symptoms								
Ellison et al., 2011 [29]	United States	Cross-sectional	*N* = 1,250; Nationwide sample of Presbyterian Church (USA) members (race and ethnicity not provided); Age: M = 59.47 years (SD = 13.48 years)	Not reported	Religious doubts, measured using a three-item index assessing doubts about faith due to evil in the world, faith-science conflicts, and meaning in life	Sleep latency measured using the Pittsburgh Sleep Quality Index (PSQI)	None reported	Religious doubts were significantly associated with increased difficulty falling asleep. These associations were partially mediated by psychological distress.
White et al., 2018 [54]	United States	Cross-sectional	*N* = 13,238; Active-duty US military personnel (race and ethnicity not available); Male (72.4%), Female (27.6%)	Not reported	Religious attendance and religious salience (measured via self-reported survey)	Sleep disturbance, measured via self-reported past-month frequency of trouble falling/staying asleep	Higher religious salience was associated with lower odds of sleep disturbance, and it moderated the relationship between combat casualty exposure and sleep disturbance.	Religious attendance was associated with lower sleep disturbance but did not moderate the impact of combat casualty exposure on sleep quality.
Bierman et al., 2018 [55]	United States	Longitudinal	*N* = 7,130; Older adults from the Health and Retirement Study (HRS); Age: M = 66.03 years (SD = 10.42 years)	Not reported	Religious attendance and personal religious importance (measured using self-reported frequency of attendance and a 4-item scale assessing personal religiosity)	Sleep problems, measured via self-reported frequency of trouble falling asleep, waking up during the night, waking up too early, and waking up feeling unrested	Religious attendance buffered the association between chronic discrimination and sleep problems among women. Women who attended religious services regularly did not show a significant association between discrimination and sleep problems.	Religious attendance did not buffer the discrimination-sleep association for men. Personal religious importance was not a significant buffer for sleep problems in either gender.
Voiß et al., 2019 [56]	United States	Cross-sectional	*N* = 26,742; Nationally representative sample; No sleep problems: Men (53.3%); Women (46.7%), Age − 18–29 years (23.4%), 30–39 years (17.98%), 40–49 years (15.8%), 50–64 years (23.3%), 65 years and up (17.7%); Sleep problems: Men (43%), Women (57%), Age − 18–29 years (18.6%), 30–39 years (16.3%), 40–49 years (16.4%), 50–64 years (28.1%), 65 years and up (20.5%)	Not reported	Mind-body medicine (MBM), including spiritual meditation, mindfulness meditation, yoga, tai chi, guided imagery, progressive relaxation	Self-reported trouble falling asleep, trouble staying asleep, and sleep duration	Individuals using MBM (including spiritual meditation) had better sleep outcomes compared to non-users. Yoga, spiritual meditation, and mindfulness meditation were the most commonly used practices associated with improved sleep.	Despite evidence supporting tai chi for sleep improvement, it was used less frequently among individuals with sleep problems. Differences in MBM use were observed across racial/ethnic groups, with non-Hispanic Whites being more likely to use MBM.
Voiss et al., 2019 [57]	United States	Cross-sectional	*N* = 26,742; Nationally representative sample	Not reported	Mind-body medicine (MBM), including spiritual meditation, mindfulness meditation, yoga, tai chi, guided imagery, progressive relaxation	Self-reported trouble falling asleep, trouble staying asleep, and sleep duration	Among cancer survivors with sleep problems, 14.3% used spiritual meditation, 17.9% used yoga, and 6.0% used mindfulness meditation. MBM use was associated with improved sleep quality.	MBM use was more prevalent among those with higher education and income, leading to disparities in accessibility. Tai chi, despite evidence for sleep improvement, was underutilized.
Macinko & Upchurch, 2019 [58]	United States	Cross-sectional	*N* = 21,274; Do not practice meditation = 63.5% Non-Hispanic White, 16.9% Hispanic, 12.5% Non-Hispanic Black, 5.9% Non-Hispanic Asian, 1.2% Other; Practice meditation = 69.2% Non-Hispanic White, 12% Hispanic, 11.2% Non-Hispanic Black, 6.7% Non-Hispanic Asian, 1.0% Other; No meditation: Male (50.73%), Age: M = 47.75 years; Meditation: Male (37.42%), Age: M = 46.15 years	Not reported	Spiritual meditation, mantra meditation, mindfulness meditation, guided imagery, progressive relaxation (self-reported)	Self-reported sleep problems (trouble falling asleep or staying asleep for two or more nights in the past week)	None reported	Meditation use was lower among older adults and racial/ethnic minorities. Sleep problems were more prevalent among individuals who reported cost/access barriers to healthcare, and these individuals were more likely to use meditation for self-care.
Hill et al., 2020 [59]	United States	Cross-sectional	*N* = 2,323; Older Mexican Americans (age 65+) from the Hispanic Established Populations for the Epidemiologic Studies of the Elderly (H-EPESE); Female (58%), Male (42%); Age: M = 73.07 years	Not reported	Religious attendance (measured using self-reported frequency of attending religious services)	Sleep disturbance, measured as a composite index of self-reported trouble falling asleep, waking up several times per night, trouble staying asleep, and waking up tired	Religious attendance was inversely associated with sleep disturbance, and depressive symptoms mediated this association.	None reported
Moradi et al., 2022 [37]	Iran	Randomized Controlled Trial	*N* = 40; Pregnant women in Abhar, Iran; Women (100%); Mean age intervention group = 26.65 years, Mean age control group = 28.55 years	Muslim	Spiritual content counseling based on Islamic teachings (8-session intervention twice a week for 4 weeks)	Insomnia severity measured using the Insomnia Severity Index (ISI)	Participants in the intervention group showed significant reductions in insomnia severity compared to the control group. The effects persisted through the second and third trimesters (*p* < 0.001).	None reported
de Diego-Cordero et al., 2022 [60]	Global (multiple countries)	Scoping Review	Review of 10 studies (samples varied across studies)	Not reported	Spiritual and religious interventions including yoga, mantra, mindfulness, prayer, meditation, daily spiritual experiences, and psycho-religious training	Insomnia symptoms and sleep quality (varied measures across studies)	Spiritual and religious interventions showed a positive influence on sleep, particularly in improving insomnia symptoms. Mindfulness, yoga, and prayer were associated with better sleep quality.	None reported
Ali et al., 2022 [61]	United States	Intervention, Pilot Study	*N* = 84; sub-group of Black congregation members in Bronx, NY (including from Caribbean as well as Central and South America); Age: M = 61.5 years (SD: 14.1 years)	Christian	Spirituality-based, peer-led intervention using an 8-session faith-based curriculum	Trouble falling/staying asleep and duration sleep, measured using the National Health and Nutrition Examination Survey’s (NHANES) sleep survey items	The intervention significantly reduced the odds of reporting trouble falling or staying asleep post-intervention (*p* = 0.0269).	No significant change in total sleep duration post-intervention.
Britt et al., 2023 [62]	United States	Cross-sectional	*N* = 72; 76.5% Non-Hispanic White, 19.8% Non-Hispanic Black, 3.7% Hispanic, older adults; Age: M = 84.23 years with all-cause dementia; Male (35.5%) & Female (64.5%)	Christian (91.3%), Jewish (5.5%), None (2.6%), Other (0.6%)	Religious service attendance (measured as frequency of attendance from self-reported survey responses)	Sleep disturbances, measured using self-reported responses to three yes/no sleep questions (difficulty falling asleep, waking frequently, waking too early)	Higher frequency of religious attendance was significantly associated with fewer sleep disturbances (Spearman’s Rho: −0.275, *p* < 0.0005).	None reported
Upenieks et al., 2022 [63]	United States	Cross-sectional	*N* = 1,404; 70% White, 30% Non-White (Hispanic, Black, Asian, American Indian, Pacific Islander, Multi-race non-Hispanic); Male (43.41%), Female (56.59%); Age: M = 54.45 years (SD = 16.97 years)	Christian (73.39%) Jewish (1.97%), Other (7.82%), No religion (16.81%)	Religious attendance, divine control, and religious doubt were measured using self-reported survey questions from the Baylor Religion Survey (BRS)	Sleep quality, measured using self-reported responses to difficulty falling asleep and feeling rested in the morning (4-point scale)	Higher religious attendance and stronger perceptions of divine control were associated with better sleep quality. The association was mediated by a greater sense of meaning and purpose in life.	Religious doubt was associated with lower sleep quality, but this association was attenuated after adjusting for a sense of meaning and purpose in life.
Dubar et al., 2024 [64]	United States	Cross-sectional	*N* = 695; 70.4% White, 10.2% Black/African American, 5.9% Asian, 3.0% Mixed race, 0.7% American Indian/Alaskan Native, 0.1% Native Hawaiian/Other Pacific Islander, 1.6% Other, 8.1% Missing; Male (22.3%), Female (77.3%) & Other (0.4%); Age: (18–29) years = 354 [50.9%], (30–49) years = 108 [15.5%], (50+) years = 233 [33.5%]	Multiple (Majority Christian [85.9%])	Religious coping (positive and negative), religious doubt, and religious support were measured using the Brief Religious Coping (RCOPE) scale	Sleep latency, insomnia symptoms measured using the Insomnia Severity Index (ISI), sleep duration (self-reported), sleep hygiene (cognitive-emotional and substance use subscales)	Secure copers, characterized by high positive and low negative religious coping, had the best sleep profiles with shorter sleep onset latency, fewer insomnia symptoms, and better sleep hygiene.	Conflicted and anxious copers, characterized by high negative religious coping, had the worst sleep profiles, with longer sleep onset latency, more insomnia symptoms, and worse sleep hygiene.
Upenieks et al., 2024 [65]	United States	Cross-sectional	*N* = 1395; 70.11% White, 29.89% Non-White (Hispanic, Black, Asian, American Indian, Pacific Islander, Multi-race non-Hispanic); Male (43.41%), Female (56.59%); Age: M = 54.45 years (SD = 16.97 years)	Christian (73.39%) Jewish (1.97%), Other (7.82%), No religion (16.81%)	Crisis of faith, which was measured using a single-item self-report in the Baylor Religion Survey (BRS)	Sleep quality (self-reported difficulty falling asleep and feeling rested in the morning)	None reported	A crisis of faith was associated with lower sleep quality, partially mediated by lower meaning and purpose in life. The negative association was stronger for men than women.
Siswanto et al., 2024 [66]	Indonesia	Randomized Controlled Trial	*N* = 44; All Muslim participants with Type 2 Diabetes Mellitus; Male (50%) & Female (50%); Age: Group 1: M = 55.27 years(SD = 7.18 years); Group 2: M = 54.4 years (SD = 5.08 years)	Muslim	Latihan Pasrah Diri (LPD), which translates to “Self Surrender Practice,” is a spiritual-based relaxation and mindfulness intervention	Sleep quality measured using the Insomnia Severity Index (ISI)	After 8 weeks of LPD practice, the intervention group had a statistically significant decrease in ISI scores (−2.58) compared to the control group (−0.04), indicating improved sleep quality (*p* = 0.003, 95% CI −4.15, −0.92).	None reported
Agarwal & Thomas, 2024 [67]	United States	Cross-sectional	Telephone interview *N* = 3,294; Postmenopausal or had hysterectomy women; 89.7% White, 4.0% Black, 1.1% Native American, 0.4% Asian, 4.9% Other; Self-administered questionnaire *N* = 2732; Female (100%); Age: M = 64 years	Christian (77.4%) Jewish (2.7%), Other/spiritual (4.4%), Unaffiliated (15.5%)	Self-reported religious affiliation (Baptist, Lutheran, Methodist, Pentecostal/Evangelical, Protestant, Catholic, Jewish, Other, Unaffiliated, Spiritual)	Trouble falling asleep & waking up during the night measured through a self-reported frequency scale	Unaffiliated and Spiritual women were less likely to experience trouble falling asleep compared to Catholic women.	Baptist women were significantly more likely to experience trouble falling asleep compared to Catholic women, but this was explained by smoking prevalence differences.
Kader et al., 2024 [68]	Canada	Cross-sectional	*N* = 588; First Nations Sleep Health Project; adults from two Cree communities in Saskatchewan, Canada; Male (44.22%), Female (55.78%); age: M = 40.03 years (SD = 15.26 years)	Not reported	Spiritual health, measured using a self-reported 5-point Likert scale (poor, fair, good, very good, excellent) as part of the Indigenous Medicine Wheel framework	Insomnia severity, measured using the Insomnia Severity Index (ISI)	Individuals with good or better self-rated spiritual health were less likely to report moderate to severe insomnia.	The association between insomnia severity and spiritual health was weaker compared to its association with physical, mental, and emotional health. There was no significant mediation effect of multimorbidity on the relationship between insomnia and spiritual health.
Biji et al., 2025 [69]	India	Cross-sectional	*N* = 398 advanced cancer patients in palliative care; Hindu (67.1%), Muslim and others; Male 50%, Female 50%; Median age 59 years (IQR 51–65 years)	Multi-faith (predominantly Hindu; includes Muslim, Christian)	Spiritual well-being using the Functional Assessment of Chronic Illness Therapy Spiritual Well-Being Expanded Scale (FACIT-Sp-Ex) including the domains of meaning, peace, faith, and relational	Sleep disturbance using the insomnia item of European Organisation for Research and Treatment of Cancer Quality of Life Questionnaire Core 30 (EORTC QLQ-C30)	Higher spiritual well-being strongly associated with better quality of life and lower insomnia and symptom burden.	None reported
Sleep disturbances								
Phillips et al., 2006 [70]	United States	Cross-sectional	*N* = 107; HIV-infected individuals (82.6% African American, 15.1% Caucasian, 2.3% Hispanic); Male (66.7%), Female (33.3%); Age: M = 40 years (SD = 7.3 years)	Not reported	Spiritual well-being, measured using the Spiritual Well-Being Scale (SWBS)	Sleep disturbance, measured using the Pittsburgh Sleep Quality Index (PSQI)	Higher existential well-being and total spiritual well-being were significantly correlated with better sleep quality (*p* < 0.05).	Religious well-being was not significantly associated with sleep quality. All participants reported significant sleep disturbances.
Cramer et al., 2016 [71]	Germany	Randomized Controlled Bicenter Trial	*N* = 54; Patients with colorectal cancer; Male (61.1%), Female (38.9%); Age: M = 68.26 years (SD = 9.69 years)	Not reported	Hatha yoga (10-week intervention, 90-minute weekly sessions)	Sleep disturbances, measured using the Pittsburgh Sleep Quality Index (PSQI)	At week 22, the yoga group showed significantly lower sleep disturbances compared to the control group (Δ= −1.08, *p* = 0.043).	No significant group differences were observed at week 10, and overall adherence to the intervention was low.
Ji et al., 2017 [72]	Hong Kong, China	Randomized Controlled Trial	*N* = 126; Adults with mild to moderate depression and sleep disturbance; Female (77.8%), Male (22.2%); Age: M = 56.14 years (SD = 9.28 years)	Not reported	Integrative Mind-Body-Spirit (I-BMS) intervention incorporating mindfulness, qigong, and spiritual reflection	Sleep disturbance, measured using the Pittsburgh Sleep Quality Index (PSQI)	I-BMS intervention significantly improved sleep quality. The reduction in depressive symptoms partially mediated the effect of I-BMS on nighttime insomnia symptoms and fully mediated the effect on daytime insomnia symptoms.	None reported
Brewer-Smyth et al., 2020 [73]	United States	Cross-sectional, Correlational Pilot Study	*N* = 13; Women living in a faith-based homeless mission (7 African American, 3 White, 1 Hispanic, 2 mixed-race); Female (100%); Age range 20–59 Years, M = 35 years	Not reported	Religiosity and spirituality measured using the Duke University Religion Index – (DUREL) and forgiveness measured using the Heartland Forgiveness Scale (HFS)	Sleep disturbance, measured using the Quality of Life in Neurological Disorders (Neuro-QoL) Version 1.0 Sleep Disturbance Short Form	Higher religiosity, spirituality, and forgiveness were significantly correlated with better sleep quality and better overall health.	Lower religiosity and forgiveness were significantly associated with poorer sleep quality and poorer health outcomes.
Mendes et al., 2023 [74]	Brazil	Descriptive and Correlational Study/Cross-sectional	*N* = 135; Palliative care patients; Men (54.8%), Women (45.2%); Age: M = 65 years (SD = 16.9 years)	Christian (88.2%), other (4.4%), without religion (7.4%)	Spiritual well-being, measured using the Functional Assessment of Chronic Illness Therapy-Spiritual Well-Being, 12-Item Version (FACIT-Sp-12)	Sleep disturbance, measured using the Edmonton Symptom Assessment Scale (ESAS)	None reported	Spiritual well-being showed a weak negative correlation with sleep disturbance (*r* = −0.109, *p* = 0.206), but the correlation was not statistically significant. Depression showed a stronger negative correlation with spiritual well-being (*r* = −0.469, *p* < 0.001).
Coleman et al., 2024 [75]	United States	Mixed-methods, cross-sectional	Survey *N* = 91; Black/African American (*n* = 22), Latina (*n* = 51), Chinese American (*n* = 18) cervical cancer survivors Interview *N* = 12; Chinese American (*n* = 5), Latina (*n* = 3), African American (*n* = 4) cervical cancer survivors; Female (100%); Age: M = 46.38 years	Not reported	Spirituality, measured using the Functional Assessment of Chronic Illness Therapy-Spiritual Well-Being, 12 Item Version, Version 4 (FACIT-SP-12 v4)	Sleep disturbance, measured using the Patient-Reported Outcomes Measurement Information System (PROMIS)	Higher spirituality was associated with lower sleep disturbance scores, indicating better sleep quality among cervical cancer survivors.	None reported
Ulutaşdemir, et al., 2025 [76]	Turkey	Phenomenological Qualitative	*N* = 33; Earthquake survivors from 11 Turkish provinces; Men (51.51%) Female (48.48%); Age: M = 30.69 years, SD = 9.22 years	Not reported	Spiritual coping during disaster; belief in religion as a coping mechanism (qualitative)	Self-reported sleep disturbances after the earthquake (qualitative)	Spiritual coping, including prayer and belief in a higher power, was perceived to support survival and adaptation.	None reported explicitly; sleep was disrupted due to disaster trauma.
Adesogan et al., 2025 [77]	United States	Cross-sectional	*N* = 692 Black adults across four waves of the Protecting Strong African American Families project; Male (50%) & Female (50%); Age- Fathers: 39.9 years (SD = 9.6 years), Mothers: 36.6 years (SD = 7.5 years)	Not reported	Religiosity and spirituality measured using the Multidimensional Measure of Religious Involvement/Spirituality (MMRS)	Sleep problems assessed using six-item sleep scale adapted from the Medical Outcomes Study (MOS) Sleep Scale	Religiosity and spirituality showed protective effects against sleep problems over time in both univariate and multivariate models.	None reported
Sleep efficiency								
Hill et al., 2025 [78]	United States	Cross-sectional	*N* = 5,168; Adults from the All of Us Research Program (95% Non-Hispanic White); Age: M = 54.78 years (SD = 15.11 years)	Not reported	Religious attendance, measured using a single-item self-reported frequency of attending religious services	Sleep efficiency, measured using wrist actigraphy (Fitbit data)	Religious attendance was positively associated with sleep efficiency (*p* < 0.01). Individuals who attended religious services more frequently exhibited better sleep efficiency.	The association between religious attendance and sleep efficiency did not vary by levels of neighborhood disorder, contradicting the hypothesis that neighborhood disorder would moderate this relationship.
Basis et al., 2025 [52]	Israel	Cross-sectional	*N* = 233; Druze adults; religious (*n* = 93), non-religious (*n* = 140); Male 28.5%, Female 71.5%; Age: M = 36.6 years (SD = 10.8 years)	Druze Faith	Religiosity and religious orientation (end, means, quest)	Sleep efficiency (diary)	Higher religiosity associated with better sleep efficiency; anxiety mediated associations	“Religion as quest” orientation was associated with poorer sleep
Sleep/Circadian timing & regularity								
Zarhin, 2023 [25]	Israel	Qualitative	*N* = 31; Midlife Israeli Muslim adults (*n* = 18) and Jewish adults (*n* = 13) who identified as religious or very religious: Men (*n* = 14), Women (*n* = 17); Age: M = 49.41 years	Muslim and Jewish	Religious observance, including prayer times, beliefs, and prescribed sleep positions (qualitative study; no specific instrument used)	Self-reported sleep timing (qualitative interviews)	None reported	Adherence to prescribed prayer times disrupted sleep duration, particularly for Muslims and Jewish men, who had to wake up early but could not always go to sleep early enough.
Irfan et al., 2025 [79]	Global (review of Muslim sleep patterns across countries/regions)	Narrative review	Review included 70 empirical studies (1988–2024) on sleep in Muslim populations/practices	Islam	Core Islamic practices, especially Ramadan fasting	Sleep schedule, duration, quality; objective and validated subjective sleep metrics across included studies	None reported	Islamic practices (especially Ramadan fasting and schedule shifts) can alter sleep timing, duration, and sometimes sleep architecture.
Zarina et al., 2025 [80]	Israel	Cross-sectional	*N* = 473 Israeli Jewish adults age 60–88 years, M = 69.0 years (SD = 5.7 years); Religious *n* = 274; Secular *n* = 199; Male 25.6%, Female 74.4%	Judaism	Religious lifestyle/observance (religious vs. secular; Sabbath observance behaviors)	Chronotype, social jetlag, sleep duration & timing; subjective sleep quality; daytime sleepiness using the Epworth Sleepiness Scale (ESS)	Religious participants had earlier chronotype, lower social jetlag, longer sleep duration on free days, and more stable sleep timing (circadian stability)	No group differences in subjective sleep quality, latency and night awakenings.
Daytime sleepiness								
Margolis & Reed, 2004 [24]	United Arab Emirates	Cross-sectional	*N* = 137 (Men = 28%; Women = 72%) pre-Ramadan, *N* = 126 (Men = 27%, Women = 73%) during Ramadan, *N* = 109 (Men = 28%, Women = 72%) post-Ramadan; Muslim medical students; Age range = 19–23 years	Muslim	Religious practices during Ramadan, including fasting, altered sleep-wake schedules, and pre-dawn prayers	Daytime sleepiness, measured using self-reported sleep hours and the Epworth Sleepiness Scale (ESS)	Students adapted to altered schedules by increasing daytime sleep, preventing a rise in daytime sleepiness.	None reported
Sleepiness/Drowsiness								
Dyer et al., 2022 [81]	United States	Qualitative	*N* = 1,284; 83% White, 7% Hispanic, 2% Black, 1.9% Asian, 6% Other/Multiracial; Male (17%) & Female (81%); Age: Range (18–89) years, M = 49.2 years	Reiki practitioners	Reiki therapy (biofield spiritual healing)	Self-reported sleep and drowsiness (qualitative responses on post-session survey)	10% of participants reported sleep and drowsiness after Reiki, describing feelings of deep relaxation and ease.	None reported
Sleep medication use								
Ellison et al., 2011 [29]	United States	Cross-sectional	*N* = 1,250; Nationwide sample of Presbyterian Church (USA) members (race and ethnicity not provided); Age: M = 59.47 years (SD = 13.48 years)	Not reported	Religious doubts, measured using a three-item index assessing doubts about faith due to evil in the world, faith-science conflicts, and meaning in life	Pittsburgh Sleep Quality Index (PSQI)-use of sleep medication	None reported	Religious doubts were significantly associated higher use of sleep medications. These associations were partially mediated by psychological distress.
Goerge et al., 2024 [27]	United States	Cross-sectional	*N* = 323; African American long-term breast cancer survivors; Age: M = 54.8 years (SD = 9.89 years)	Not reported	Spirituality, measured using the Functional Assessment of Chronic Illness Therapy Spiritual Well-Being 12 (FACIT-Sp-12)	Pittsburgh Sleep Quality Index (PSQI) and self-reported questionnaire data	None reported	Higher spirituality scores were inversely associated with sleep medication use (aOR: 0.45, 95% CI: 0.24–0.84), suggesting that those with greater spirituality were less likely to use sleep medication.
Sleep position								
Zarhin, 2023 [25]	Israel	Qualitative	*N* = 31; Midlife Israeli Muslim adults (*n* = 18) and Jewish adults (*n* = 13) who identified as religious or very religious; Men (*n* = 14), Women (*n* = 17); Age: M = 49.41 years	Muslim and Jewish	Religious observance, including prayer times, beliefs, and prescribed sleep positions (qualitative study; no specific instrument used)	Self-reported sleep position (qualitative interviews)	Participants became accustomed to sleeping in prescribed religious positions, which they found beneficial.	None reported

Given the central role that race and ethnicity play in shaping social experiences in the United States [82, 83], and prior evidence that racial and ethnic groups differ in religiosity/spirituality and sleep health [22, 23], we summarized how race and ethnicity were reported across U.S. based studies. Among included U.S. investigations, a few (*n* = 4) used nationally representative samples, while most relied on regional, clinic-based, or cohort-specific populations. Many (*n* = 9) U.S. studies included predominantly White samples, and a notable proportion did not report race or ethnicity in sufficient detail to characterize sample composition. Some studies (*n* = 5) focused explicitly on a single racial or ethnic group, most commonly Black/African American adults, whereas others included racially and ethnically diverse samples but did not examine differences by race or ethnicity.

### Religious Context of Samples

Religious traditions were variably reported across studies. Nearly half (44%) of studies did not specify participants’ religion. Among the 56% that did, Christianity or majority-Christian populations were most frequently represented (24%); in several of these majority-Christian samples, Jewish individuals were also included in small numbers and, when reported, typically comprised a very small proportion of the sample. Within these samples, Jewish individuals were rarely examined as a distinct analytic group. Islam was represented in 24% of studies. One study focused on Judaism, one included both Islamic and Jewish participants, one examined the Druze faith, and one included a predominantly Hindu sample. Other world religions, such as Buddhism, Taoism, or indigenous and traditional spiritual systems, were not represented in the identified literature. Moreover, few studies explicitly compared sleep outcomes across religious traditions, limiting the ability to assess whether particular practices or belief systems are differentially associated with sleep health.

### Religion and Spirituality in Relation to Sleep Health

#### Sleep Duration

Religious and spiritual factors appear to influence sleep duration through a combination of psychological and behavioral mechanisms, but findings vary by population and practice. In a study of African American breast cancer survivors, greater spirituality scores were associated with longer sleep duration, yet participants who reported higher spirituality were also more likely to use sleep medications, suggesting that faith may support coping but does not necessarily eliminate the need for medical management of sleep [27]. Qualitative work among Israeli Muslim and Jewish individuals offered additional nuance: participants emphasized that deep faith provided a sense of peace that they believed facilitated rest, but early morning prayer observance shortened total nocturnal sleep, demonstrating how religious ritual can create both benefits and constraints for sleep [25]. In U.S. population-based data, religious service attendance was initially associated with longer sleep duration, but the association weakened after accounting for psychosocial and socioeconomic factors (some of which may have been mediating factors), underscoring the challenge of disentangling religious influences from broader social determinants [26]. Earlier research during Ramadan demonstrated that fasting and altered daily routines reduced nighttime sleep while increasing daytime rest, highlighting how religious observance can shift the distribution rather than the total amount of sleep [24]. Together, these findings indicate that R/S may shape sleep duration in complex ways, sometimes fostering longer rest and other times constraining it through ritual demands.

#### Sleep Quality

Sleep quality has been examined across diverse populations, and R/S are most consistently associated with better perceived sleep, though the strength and direction of associations depend on the type of engagement and the context [32]. In a national cohort of older African Americans, frequent service attendance was associated with fewer reports of restless sleep, while passive religious television viewing was associated with worse sleep quality, suggesting that active participation may be more beneficial than passive consumption [38], potentially in part due to greater exposure to light at night from sleeping with the television on, which has been associated with poorer sleep across multiple dimensions [84]. In another U.S. study, secure attachment to God and assurance of salvation were associated with higher self-reported sleep quality, whereas religious doubt and conflict were linked to poorer outcomes [35]. Among complementary medicine users, engagement with Reiki and biofield spiritual healing was associated with improved rest and relaxation [81]. In a review of population-based studies, religious involvement was generally associated with higher subjective sleep quality, but religious doubts were consistently associated with poorer sleep outcomes [18].

Evidence outside the U.S. adds further support. Hemodialysis patients in Taiwan who reported greater religious activity also reported better sleep [28]. In Indonesia, higher spiritual well-being was correlated with better rest [36]. In Iran, multiple studies reported that higher spiritual well-being was positively associated with sleep quality across clinical and community populations [30, 31, 37, 39–41]. Religious or spiritual resources were associated with improved sleep in Brazilian nursing samples [45]. In a meta-analysis, Managing Cancer and Living Meaningfully (CALM) therapy, a psycho-oncology intervention that integrates existential and spiritual components, was associated with significant improvements in sleep quality at approximately 3-month follow-up compared with usual care [42]. In Korean oncology care, an 8-week spiritually oriented mind-subtraction meditation program was associated with significant improvements in subjective sleep quality as indicated by Pittsburgh Sleep Quality Index (PSQI) scores at post-intervention; however no extended follow-up was reported [33].

Recent quasi-experimental studies further extend this evidence, demonstrating that structured spiritual care interventions, such as Benson’s spiritual relaxation combined with lavender aromatherapy, delivered twice daily (10–15 min per session), five days per week during the study period (April – August 2024), significantly improved sleep quality at post-intervention among hypertensive patients in Indonesia; however, sustained effects were not assessed beyond the treatment period [48]. Similarly, randomized trials among hemodialysis patients in Iran and Indonesia reported that spiritual care programs incorporating prayer, dhikr, or guided spiritual reflection compared with usual care led to significant improvements in sleep quality at post intervention (approximately 2–4 weeks), although longer-term follow-up was not conducted [49, 53].

Group-based spiritual reminiscence therapy has also been shown to improve global sleep quality immediately following six sessions delivered over three weeks, as well as specific PSQI domains, including sleep latency, sleep duration, and sleep disturbances among postmenopausal women in Iran [50]. A systematic review and meta-analysis of studies conducted in Iran also synthesized evidence indicating associations between spiritual health–based interventions and improved sleep quality [47]. Beyond explicitly religious interventions, a recent systematic review and meta-analysis showed that mind–body therapies with spiritual or contemplative components (e.g., yoga, mindfulness, tai chi, qigong) were associated with improved sleep quality in midlife and older women [51].

Nonetheless, not all studies showed beneficial associations. In some U.S. cohorts, religious attendance and private practices were unrelated to sleep quality once sociodemographic and psychosocial factors were considered [35, 46]. Among Israeli Druze adults, higher overall religiosity was associated with better sleep quality, whereas a quest-oriented religious orientation, characterized by religious doubt and searching, was associated with poorer sleep quality, highlighting within-faith heterogeneity [52]. Authors of a recent global review concluded that frequent attendance did not always translate into better rest, particularly when religious doubt was high, and emphasized heterogeneity in the evidence base, highlighting the need for stronger designs [43].

#### Insomnia Symptoms

Insomnia has been studied in a range of populations, with consistent evidence that supportive R/S engagement is associated with fewer symptoms, while spiritual struggle is associated with more. In the U.S., secure religious coping profiles were associated with fewer insomnia symptoms, whereas conflicted or anxious coping was associated with more symptoms [64]. Spiritual struggle and crises of faith were associated with difficulty initiating and maintaining sleep [62, 65]. Other U.S. studies similarly demonstrated that religious doubt and conflict were associated with more insomnia symptoms, whereas positive engagement was associated with fewer insomnia symptoms [29, 54, 55, 59].

Additionally, in Indonesia, an 8-week Latihan Pasrah Diri, a spiritual self-surrender practice, reduced insomnia severity measured post-intervention in adults with type 2 diabetes, although durability beyond the intervention period was not assessed [66]. In Iran, spiritual well-being was associated with fewer symptoms, while in Spain, rigid or conflictual religious experiences were associated with more symptoms [37, 60]. Among midlife and older women in the US, religious affiliation and engagement were associated with lower odds of insomnia [67]. In a study of adults with advanced cancer receiving palliative care in India, higher spiritual well-being was associated with lower insomnia symptoms and overall symptom burden [69].

In contrast, fewer (*n* = 4) U.S.-based studies reported null or context-dependent associations. In one study, religious or spiritual engagement was associated with improvements in insomnia severity but not with other sleep dimensions, such as sleep duration, indicating outcome-specific effects [61]. In another study of U.S. cancer survivors, use of mind–body or spiritually oriented practices was more common among individuals with greater symptom burden, suggesting that engagement may reflect coping in response to poor sleep rather than a protective effect [57]. Supporting this possibility of coping being a driver of observed associations, a review of global studies concluded that positive coping and meaning-making were most often associated with fewer symptoms, while spiritual struggle and doubt were consistently associated with more insomnia [43]. A population-based study further found no significant association between meditation or related practices and insomnia symptoms after accounting for demographic factors, highlighting that engagement patterns may vary by age and population rather than efficacy [58].

#### Sleep Disturbances

Sleep disturbances, including restless sleep and daytime complaints, have been studied in clinically and psychosocially vulnerable populations, often with evidence of benefits from R/S engagement. In U.S. cancer survivors, higher spirituality was associated with fewer disturbances [75]. Women living in a faith-based homeless mission reported that higher R/S and greater dispositional forgiveness, which refers to an individual’s general tendency to be forgiving, were associated with fewer sleep disturbances, while lower religiosity and forgiveness were associated with more complaints of sleep disturbances [73]. Among Turkish nurses, stronger spiritual well-being was associated with less disrupted sleep [76].

On the other hand, among palliative care patients in Brazil, associations between spiritual well-being and sleep disturbance were weak or variable [74]. In Germany and China, spiritual concerns and distress were associated with worse sleep outcomes [71, 72]. In U.S. patients with HIV, existential well-being (i.e., meaning, purpose, life satisfaction, sense of coherence) was associated with fewer sleep complaints, whereas religious well-being was not, which suggests that meaning and purpose may be more closely related to rest than institutional religiosity [70], although religiosity is often considered a source of greater meaning and purpose, especially among minoritized populations [8, 85].

#### Sleep Efficiency

Few studies have examined sleep efficiency using objective sleep outcomes. In the U.S. All of Us Research Program, religious service attendance was associated with higher actigraphy-measured sleep efficiency after accounting for multiple covariates [78]. Reviews emphasize the limited number of studies that include objective outcomes and recommend the integration of actigraphy and polysomnography in future work [18, 43].

#### Circadian Timing/Regularity

Few studies have explicitly examined circadian timing and regularity in relation to religious or spiritual practices. In a cross-sectional study of older Israeli Jewish adults, religious observance, particularly Sabbath practices, was associated with earlier chronotype, greater sleep–wake regularity, and reduced social jetlag compared with secular peers, suggesting enhanced circadian stability among religious participants [80]. Reviews of sleep among Muslim populations similarly highlight the role of religious practices, including daily prayer schedules and Ramadan fasting, in shaping sleep timing and circadian alignment, with evidence of delayed bedtimes, altered sleep–wake patterns, and context-dependent circadian misalignment [79]. Importantly, religious practices may influence circadian rhythms through multiple pathways. For example, early morning prayer may advance wake timing and increase exposure to morning light, the primary circadian zeitgeber [86], which could promote earlier chronotype and greater circadian alignment. Conversely, nighttime observances, extended evening services, or late-night rituals may delay bedtime, prolong exposure to artificial light at night, or shift the timing of light offset, thereby influencing circadian phase and sleep opportunity. Together, these examples illustrate the broader interplay between religious practices, light exposure, and chronotype, which may either support circadian stability or contribute to misalignment depending on behavioral and environmental context.

In summary, findings across sleep dimensions revealed heterogeneous but interpretable patterns. Across studies of sleep duration, quality, insomnia symptoms, disturbances, efficiency, and circadian timing, internal spiritual resources, such as spirituality, spiritual well-being, meaning, peace, and secure religious coping, were more consistently associated with better subjective sleep outcomes [27, 30, 31, 34, 63, 68, 69, 77]. In contrast, religious doubt, spiritual struggle, crises of faith, and conflictual religious orientations were associated with poorer sleep quality and greater insomnia-related symptoms [18, 29, 52, 65]. Findings for external religious practices, including service attendance and ritual observance, were more variable and often attenuated after adjustment for psychosocial factors [26, 35]. In some contexts, religious practices also influenced sleep timing and opportunity, such as Ramadan-related schedule changes or early morning prayer routines that curtailed nocturnal sleep or altered sleep–wake patterns [24, 25, 79].

### Religion/Spirituality, Sleep, and Aging

Religion/spirituality and sleep intersect across the life course; however, to date, empirical investigations of R/S–sleep associations have most frequently been conducted in midlife and older adult populations. Older adults were well represented in the literature linking religion/spirituality (R/S) with sleep, with multiple population-based and intervention studies conducted in samples aged 50 years and older [38, 55, 59, 80]. Across these studies, patterns observed in age-diverse samples generally held in later life: positive religious engagement and spiritual well-being were associated with better subjective sleep quality and fewer insomnia symptoms, whereas religious doubt and spiritual struggle were associated with poorer sleep outcomes.

Some investigations also suggested that structured religious practices may have been particularly relevant for circadian regulation in older adults. For example, religious observance was associated with earlier chronotype and greater sleep–wake regularity in adults aged 60–88 years [80], raising the possibility that routine-based practices reinforced circadian stability in later life.

These findings were noteworthy given normative age-related changes in sleep architecture and circadian timing, including increased sleep fragmentation, reduced slow-wave sleep, and a tendency toward phase advancement [6]. Psychological and social resources derived from R/S, such as meaning-making, social integration, and structured daily routines [8], may therefore have interacted with age-related physiological changes in ways that differed from younger adulthood.

However, despite strong representation of older samples, studies generally adjusted for age statistically rather than formally testing age as a moderator. As a result, it remains unclear whether R/S–sleep associations were stronger, weaker, or qualitatively different across age and especially across all developmental stages. Future longitudinal and multi-cohort research should explicitly examine age-related heterogeneity to clarify whether R/S engagement differentially influenced sleep health across the life course.

### Conceptual Distinction Between Religiosity and Spirituality

Across sleep outcomes, heterogeneity in findings appears partly attributable to differences in how religious and spiritual engagement are operationalized. Specifically, studies that emphasize internal spiritual resources (e.g., meaning, purpose, or existential well-being, i.e., the outcomes of religiosity) [9] tend to report more consistent associations with better sleep quality and fewer insomnia symptoms, whereas studies focused on external religious practices (e.g., service attendance or ritual observance) [7, 8] show more variable associations that often attenuate after accounting for social and psychological covariates/mediators [35, 37]. In other contexts, external practices may even limit sleep opportunity, such as early morning prayer obligations in Islam that curtail nocturnal rest [25]. Understanding both dimensions is essential to interpreting the mixed evidence in the literature.

### Potential Mechanisms Underlying Pathways between R/S and Sleep

Several pathways may plausibly connect R/S with sleep health. Social and environmental factors provide an important insight into how religion and spirituality may influence sleep. Religious congregations and spiritual communities can provide social integration, belonging, and tangible and emotional support, which help individuals cope with stressors that are known to disrupt sleep [7, 8, 87]. These networks may also reinforce health-promoting norms such as regular routines and discouragement of substance use, which indirectly support sleep health [18]. Social support derived from religious engagement has been shown to buffer psychological distress, reduce loneliness, and improve coping in populations managing chronic illness and life stress, all of which are relevant for sleep quality [88–90]. Qualitative research among Māori populations further demonstrates that cultural worldviews shape the meaning of sleep itself, with participants describing sleep as a spiritually active state in which the *wairua* (spirit) awakens to provide guidance, opportunities for learning, and a sense of peace and purpose [91]. Such perspectives highlight that the relationship between religion, spirituality, and sleep extends beyond individual behaviors or coping processes, underscoring the importance of incorporating culturally grounded frameworks alongside biomedical models.

At the same time, the benefits of religious community are not universal and may be influenced by environmental conditions. In disadvantaged neighborhoods, the stress of disorder and insecurity may interact with religious and spiritual struggles in ways that compromise well-being. Individuals who reported divine struggles, such as feeling punished or abandoned by God, in the context of neighborhood disorder exhibited greater psychological distress, suggesting that broader social and environmental stressors may amplify the adverse aspects of religious involvement [78]. This highlights the importance of considering how environmental exposures and social context shape whether religion and spirituality act as protective or risk factors for sleep.

Beyond these contextual influences, religion and spirituality may shape sleep through everyday behaviors and routines [56]. Religious teachings often discourage alcohol, tobacco, and other substances that disrupt sleep physiology, and many traditions promote structured routines and contemplative practices, such as prayer and meditation, that may serve as consistent pre-sleep rituals and reduce arousal [7, 8, 18]. Regular participation in ritual practices may also anchor daily structure and reinforce behavioral consistency, which can support sleep continuity and promote consistent wake timing that stabilizes circadian rhythms [80]. Conversely, as indicated earlier, certain religious obligations can limit sleep opportunity [25]. Obligatory early rising, extended evening services, or nighttime observances may reduce sleep opportunity or contribute to chronic sleep restriction when not offset by earlier bedtimes [24, 79]. Empirical evidence from religious populations further suggests that religiosity may operate through health-related self-regulation pathways, including dietary self-efficacy and adherence to health-promoting behaviors, which could indirectly influence sleep through metabolic, inflammatory, and behavioral mechanisms [92]. Importantly, evidence also points to bidirectional influences [44]. Individuals with better sleep quality and adequate duration may be more likely to donate to local charities and places of worship, suggesting that sleep and religious or spiritual engagement may mutually reinforce one another through shared social and emotional processes [93].

Psychological processes are also central in explaining how religion and spirituality influence sleep. Religious and spiritual engagement often provides a sense of meaning, purpose, and existential security, which can buffer against stress, worry, and hyperarousal, factors strongly implicated in insomnia and poor sleep quality [7, 8, 18]. For instance, a crisis of faith was associated with poorer sleep quality largely through diminished sense of meaning, underscoring the role of existential well-being as a protective psychological resource [65]. Similarly, doubts about salvation and conflict with religious beliefs were associated with more symptoms of anxiety and poorer self-reported sleep outcomes, providing evidence that unresolved spiritual conflict may intensify psychological distress and disrupt rest [29]. Meta-analytic evidence further indicates that positive religious coping, such as seeking spiritual support or reframing stress as an opportunity for growth, is moderately associated with better psychological adjustment, while negative religious coping, including spiritual discontent or perceiving punishment from God, is reliably associated with poorer adjustment [94]. Together, these findings illustrate that psychological dimensions of religion and spirituality can either mitigate or exacerbate stress-related processes that are central to sleep regulation.

Finally, biological pathways may link religious and spiritual engagement to sleep-related physiology. Biologically, R/S engagement has been associated with neuroendocrine and immune processes. In a meta-analysis of religiosity, spirituality, and physiological health markers, small but significant beneficial associations were observed with cardiovascular and immune function, including lower blood pressure, healthier lipid profiles, reduced C-reactive protein, and improved immune markers, which supports the idea that religion and spirituality may influence sleep indirectly through biological pathways that regulate stress and inflammation [95]. Moreover, in a study of postmenopausal women from the U.S. Nurses’ Health Study II, religious and spiritual struggles were associated with higher daily cortisol output, while greater religious or spiritual coping was associated with a slower nighttime rise in cortisol [96]. Non-theistic daily spiritual experiences such as feelings of inner peace or connection to nature were associated with a slower nighttime rise in dehydroepiandrosterone (DHEA), an adrenal hormone thought to counterbalance cortisol’s effects on the stress response, and with higher cortisol-to-DHEA ratios at both waking and bedtime [96].

### Limitations and Gaps in the Literature

Although growing evidence suggests R/S is associated with sleep health, the existing literature has several important limitations. First, most studies rely on cross-sectional designs, which restricts the ability to draw causal inferences about the directionality of associations. Even among intervention studies, sleep outcomes were typically assessed immediately post-intervention or within short-term follow-up periods (e.g., several weeks to three months). As a result, it remains unclear whether observed improvements in sleep are durable or attenuate over time. Longitudinal and experimental studies with extended follow-up are needed to clarify directionality, assess potential shared underlying determinants, and determine whether intervention effects are sustained over time.

Second, research has largely been confined to adult populations, with no studies identified among children or adolescents. Considering that sleep undergoes developmental changes across the lifespan, and that religious and spiritual socialization begins early in life, extending this research to younger populations is an important gap. The absence of youth-focused studies also limits understanding of how R/S processes interact with rapidly shifting sleep timing, school demands, family practices, and identity development.

Third, measurement challenges further complicate interpretation. Many studies relied on single-item indicators of religiosity (e.g., frequency of service attendance) or sleep (e.g., a global quality rating), and often used the terms religion and spirituality interchangeably without differentiating external practices from internal dimensions such as meaning and purpose. Such approaches fail to capture the multidimensionality of either construct and contribute to variability across findings, making it difficult to determine whether observed associations reflect social integration, existential resources, behavioral regulation, or other underlying mechanisms. Future studies should use validated instruments that clearly distinguish religiosity as external practices from spirituality as internal meaning (without contamination with indicators of mental health), and that assess sleep across multiple domains. Moreover, most research has relied on self-reported sleep outcomes, with limited integration of objective measures such as actigraphy or polysomnography; incorporating objective assessments would strengthen mechanistic inference by linking R/S more directly to physiological sleep processes.

Fourth, the literature has also disproportionately emphasized positive aspects of R/S while underrepresenting negative experiences. Few studies have examined religious or spiritual struggles, doubt, or perceptions of divine punishment (often inversely associated with religious involvement), despite evidence that such factors are associated with greater distress and poor sleep health. Similarly, few studies stratified results by sex, by religious tradition, or by race and ethnicity in the US context, and nearly half of studies did not report participants’ religious affiliation. This lack of granularity restricts the ability to examine whether certain religious traditions or demographic groups benefit more from positive R/S engagement or are more vulnerable to its negative dimensions.

Fifth, research is geographically narrow. Most studies were conducted in North America and Southwest Asia, with very few in Latin America and none in the African region. Although the connection between religious beliefs and sleep was acknowledged by a literature review regarding sleep medicine in Africa [97], the absence of empirical studies from the African region is particularly notable given that many sub-Saharan African countries are home to some of the world’s highest rates of religious affiliation, with Christians and Muslims together accounting for approximately 95% of the population [16]. Without data from African populations, the field lacks insights into culturally rooted practices that may influence sleep in ways not captured in Western or Asian contexts. This geographic imbalance limits the generalizability of current findings and constrains understanding of how R/S-sleep associations operate across the full range of global faith traditions and cultural environments.

Finally, future research should include youth and also explicitly examine bidirectional pathways between sleep and religiosity/spirituality. While most existing studies have focused on how R/S relates to sleep outcomes, far less attention has been given to whether sleep disruption, chronic sleep deprivation, or sleep disorders (e.g., insomnia, sleep fragmentation, sleep apnea) contribute to changes in religious or spiritual cognition, coping, or meaning-making, particularly in older adulthood, when nocturnal awakenings and sleep-related distress become more common [6]. In addition, mechanistic investigations should incorporate REM-related phenomena, such as sleep paralysis and hypnagogic or hypnopompic hallucinations, which have been interpreted in some cultural contexts as spiritual or supernatural experiences [98]. Although such phenomena were not assessed in the studies included in this review, examining how nocturnal neurophysiology intersects with religious interpretation represents an important and understudied direction for future research.

## Conclusions

In summary, literature to date reveals that positive dimensions of religion and spirituality (R/S) such as meaning, coping, and social support were generally associated with better sleep, while struggles and doubt were associated with poorer sleep health. Yet much of the evidence is based on cross-sectional, self-reported data from limited populations. These findings have important public health implications, highlighting the need to consider R/S as potential psychosocial influences on sleep within both research and practice. For instance, in clinical and community settings, integrating awareness of spiritual coping, daily practices, and belief-related stress may enhance person-centered approaches to promoting healthy sleep. Future studies that employ longitudinal and experimental designs, validated multidimensional measures, and objective sleep assessments in diverse cultural and faith contexts will be critical to advancing this field and strengthening the scientific foundation needed to inform culturally responsive interventions aimed at improving sleep health at the population level.

## Key References

- Hill TD, Deangelis R, Ellison CG. Religious involvement as a social determinant of sleep: an initial review and conceptual model. Sleep Health*.* 2018;4(4):325–330.
  - ○ This review introduced religion as a social determinant of sleep and outlined key pathways linking religious involvement to sleep outcomes, providing foundational conceptual framing for the current literature.
- Koenig HG, VanderWeele T, Peteet JR. Handbook of Religion and Health. 3rd ed. Oxford University Press; 2024.
  - ○ This comprehensive reference synthesizes definitions, measurement, and mechanisms linking religiosity and spirituality to health outcomes, grounding the conceptual distinction and measurement issues discussed in this review.

## Supplementary Information

Below is the link to the electronic supplementary material.

Supplementary Material 1 (PDF 57.4 KB)

## Data Availability

No datasets were generated or analysed during the current study.
