# Supplementary material for: Religion and Spirituality as Social Determinants of Sleep Health Across the Globe: A Narrative Review
Source: Curr Sleep Med Rep. 2026 May 19;12(1):26. doi: 10.1007/s40675-026-00374-y (PMC13186892; doi:10.1007/s40675-026-00374-y)
Supplement: Supplementary file 1 — Supplementary Material 1 (PDF 57.4 KB) [file 40675_2026_374_MOESM1_ESM.pdf]

Supplemental table 1. Comprehensive list of librarian-generated search terms for each databases used for the literature review.

Databases	Search Strings
PubMed	("Sleep"[Mesh] OR "Dyssomnias"[Mesh] OR "Sleepiness"[Mesh] OR "Wakefulness"[Mesh] OR "Snoring"[Mesh] OR sleep*[tiab] OR asleep[tiab] OR somn*[tiab] OR awake*[tiab] OR waking[tiab] OR awoke*[tiab] OR woke*[tiab] OR wakefulness[tiab] OR insomnia[tiab] OR hypersomn*[tiab] OR drows*[tiab] OR dyssomnia*[tiab] OR parasomnia*[tiab] OR napping[tiab] OR naps[tiab] OR siesta*[tiab] OR polysomnograph*[tiab] OR "Sleep Aids, Pharmaceutical" [Pharmacological Action] OR ambien[tiab] OR zolpidem[tiab] OR dalmane[tiab] OR flurazepam-hydrochloride[tiab] OR halcion[tiab] OR triazolam[tiab] OR lunesta[tiab] OR eszopiclone[tiab] OR prosom[tiab] OR estazolam[tiab] OR restoril[tiab] OR temazepam[tiab] OR rozerem[tiab] OR ramelteon[tiab] OR silenor[tiab] OR doxepin[tiab]) AND (“Religion”[Mesh] OR “Religious Philosophies”[Mesh] OR “Spiritual Therapies”[Mesh] OR “Spirituality”[Mesh] OR “Mind-Body Therapies"[Mesh] OR religio*[tiab] OR anthroposophy[tiab] OR Christianity[tiab] OR Catholicism[tiab] OR Christian Science[tiab] OR Church of Jesus Christ of Latter-day Saints[tiab] OR Eastern Orthodoxy[tiab] OR Jehovah’s Witnesses[tiab] OR Protestantism[tiab] OR Saints[tiab] OR Hinduism[tiab] OR Islam[tiab] OR Muslim[tiab] OR Judaism[tiab] OR religious philosophies[tiab] OR Taoism*[tiab] OR Confucianism[tiab] OR Mysticism[tiab] Spiritual*[tiab] OR theology[tiab] OR church*[tiab] OR faith healing[tiab])
Web Of Science	TI= (sleep* OR asleep OR somn* OR “frequent waking” OR “night waking” OR awoke* OR wakefulness OR insomnia OR hypersomn* OR drows* OR dyssomnia* OR parasomnia* OR napping OR naps OR siesta* OR polysomnograph* OR ambien OR zolpidem OR dalmane OR “flurazepam-hydrochloride” OR halcion OR triazolam OR lunesta OR eszopiclone OR prosom OR estazolam OR restoril OR temazepam OR rozerem OR ramelteon OR silenor OR doxepin) AND

	TI=("Religious Philosophies" OR Spirit* OR "Mind-Body Therapies" OR religio* OR anthroposophy OR Christianity OR Catholicism OR "Christian Science" OR "Church of Jesus Christ of Latter-day Saints" OR "Eastern Orthodoxy" OR "Jehovah's Witnesses" OR Protestantism OR Saints OR Hinduism OR Islam OR Judaism OR Taoism* OR Confucianism OR Mysticism Spiritual* OR theology OR church* OR "faith healing")
Embase	('sleep'/exp OR sleep*:ti,ab,kw OR asleep:ti,ab,kw OR somn*:ti,ab,kw OR awake*:ti,ab,kw OR waking:ti,ab,kw OR awoke*:ti,ab,kw OR woke*:ti,ab,kw OR wakefulness:ti,ab,kw OR insomnia:ti,ab,kw OR hypersomn*:ti,ab,kw OR drows*:ti,ab,kw OR dyssomnia*:ti,ab,kw OR napping:ti,ab,kw OR siesta*:ti,ab,kw OR nightmare*:ti,ab,kw OR polysomnograph*:ti,ab,kw OR ambien:ti,ab,kw OR zolpidem:ti,ab,kw OR dalmane:ti,ab,kw OR flurazepam-hydrochloride:ti,ab,kw OR halcion:ti,ab,kw OR triazolam:ti,ab,kw OR lunesta:ti,ab,kw OR eszopiclone:ti,ab,kw OR prosom:ti,ab,kw OR estazolam:ti,ab,kw OR restoril:ti,ab,kw OR temazepam:ti,ab,kw OR rozerem:ti,ab,kw OR ramelteon:ti,ab,kw OR silenor:ti,ab,kw OR doxepin:ti,ab,kw) AND ('Spiritual healing'/exp OR religion/exp OR religio*:ti,ab,kw OR spiritual*:ti,ab,kw OR anthroposophy:ti,ab,kw OR Christianity:ti,ab,kw OR Catholicism:ti,ab,kw OR 'Christian Science':ti,ab,kw OR 'Church of Jesus Christ of Latter-day Saints':ti,ab,kw OR 'Eastern Orthodoxy':ti,ab,kw OR 'Jehovahs Witnesses':ti,ab,kw OR Protestantism:ti,ab,kw OR Saints:ti,ab,kw OR Hinduism:ti,ab,kw OR Islam:ti,ab,kw OR Judaism:ti,ab,kw OR Taoism*:ti,ab,kw OR Confucianism:ti,ab,kw OR Mysticism Spiritual*:ti,ab,kw OR theology:ti,ab,kw OR church*:ti,ab,kw OR 'faith healing':ti,ab,kw)
